# The Feasibility of the Adverse Childhood Experiences Questionnaire among Women in Danish Antenatal Care: A Mixed-Methods Study

**DOI:** 10.3390/ijerph20166601

**Published:** 2023-08-18

**Authors:** Helle Johnsen, Mette Juhl, Eva Rydahl, Sara Mbaye Karentius, Sabine Marie Rath, Majbritt Friis-Alstrup, Mette Grønbæk Backhausen, Katrine Røhder, Michaela Louise Schiøtz, Lotte Broberg, Vibeke de Lichtenberg

**Affiliations:** 1 Department of Midwifery and Therapeutic Sciences, University College Copenhagen, Sigurdsgade 26, 2200 Copenhagen, Denmark; 2 Department of Gynaecology and Obstetrics, Holbæk Hospital, Smedelundsgade 60, 4300 Holbæk, Denmark; samj@regionsjaelland.dk; 3 Department of Obstetrics, Slagelse Hospital, Fælledvej 13, 4200 Slagelse, Denmark; mfu@regionsjaelland.dk; 4 Department of Gynaecology and Obstetrics, Zealand University Hospital Roskilde, Sygehusvej 10, 4000 Roskilde, Denmark; mgb@regionsjaelland.dk; 5 Department of Psychology, Copenhagen University, Øster Farimagsgade 2A, 1350 Copenhagen, Denmark; katrine.rohder@psy.ku.dk; 6 The Family Clinic, Department of Obstetrics and Gynaecology, Amager and Hvidovre Hospital, Pavillon 4, Østre Hospitalsvej 5A, 2650 Hvidovre, Denmark; 7 Center for Clinical Research and Prevention, Bispebjerg and Frederiksberg University Hospital, The Capital Region of Denmark, Nordre Fasanvej 57, 2000 Frederiksberg, Denmarklotte.broberg.01@regionh.dk (L.B.)

**Keywords:** adverse childhood experiences, antenatal care, pregnancy, screening

## Abstract

A traumatic upbringing increases the risks of antenatal health problems, unfavourable pregnancy outcomes, and mental disorders. Such childhood experiences may affect women’s pa-renting skills and the social–emotional functioning of their children. Research on screening for adverse childhood experiences in antenatal care is limited. The objective of this study was to explore pregnant women’s attitudes towards and experiences of an adverse childhood experiences questionnaire, and to assess the relevance of the questionnaire among a population of pregnant women referred to antenatal care levels one and two, targeting women who are generally not perceived to be vulnerable. Data were collected at three maternity wards and consisted of quantitative data on 1352 women’s adverse childhood experience scores, structured observations of 18 midwifery visits, and in-depth interviews with 15 pregnant women. Quantitative data were analysed by descriptive statistics, and qualitative data were analysed using systematic text condensation. The qualitative analysis revealed two main categories: “Being screened for childhood adversities” and “Having adverse childhood experiences”. In the study population, the prevalence of adverse childhood experiences was high. The women assessed the adverse childhood experiences questionnaire to be a relevant and acceptable screening method. Furthermore, women’s perceptions of their relationship with their midwife greatly impacted their attitudes towards and experiences of the questionnaire.

## 1. Introduction

Adults with adverse childhood experiences (ACEs: abuse, neglect, and household dysfunction) are at increased risk of having poorer self-perceived health, mental illness, and cardiovascular disease, compared to adults without ACEs [1,2,3,4,5]. The number of ACEs has been associated with poorer health and lower educational attainment [3,6,7,8]. A recent review estimated that in European countries, 23.5% of adults have one ACE and 18.7% have two ACEs or more [7]. Similar proportions of ACEs have been found between high-, high–middle-, and low–lower-middle-income countries [9].

Studies have shown that having one or two ACEs is associated with a higher risk of being in a relationship with domestic violence [10] and having at least two ACEs is associated with a higher risk of having an unwanted pregnancy [11]. Regarding reproduction, women with ACEs are more likely to enter pregnancy with a chronic health condition [12], to have past obstetrical risk factors and problems in the present pregnancy [13], and to give birth preterm [14]. Mothers with four ACEs or more have a twofold greater risk of experiencing biomedical risks (e.g., low birth weight, short gestation, and need for intensive care) and a fivefold increased risk of experiencing psychosocial risks (e.g., single or teen motherhood and maternal low education) [15]. Women with ACEs are also more likely to experience psychosocial difficulties during pregnancy or after birth, such as depression or anxiety [10,12,13,14,16,17,18]. The negative health consequences associated with having ACEs may be buffered by social support such as living with the baby’s father or a partner, as well as emotional, informational, and material support [13,17]. Resilience, including the ability to bounce back after hardship, handle unpleasant or painful feelings, and adapt to change, can also serve as a buffer [10,19]. 

Parents with ACEs are more likely to experience stress after birth [20] and to have negative parenting behaviours [21], which may affect their child’s early development. Parental ACEs have been found to be an important predictor of unfavourable outcomes in children, and they are associated with delayed early child development, affecting motor skills, language, social interaction, mood, behaviour, and overall health status negatively [22]. These conditions may also increase the risk of insecure attachment patterns in the offspring, and in more serious cases they can lead to disorganised attachment [23]. The direct effects of parental ACEs are often mediated or moderated by individual and/or fa-mily factors [22]. Studies show that in families with multiple problems, the risk of insecure attachment patterns in the offspring—especially disorganised attachment—is significantly increased [23]. In addition, children with insecure attachment patterns may replicate parental behaviour as adults when caring for children of their own, which may subsequently impact child development [24,25].

According to the World Health Organization (WHO), antenatal care plays a pivotal role in promoting healthy mothers and thriving families [26]. The WHO also highlights the significance of a traumatic upbringing for lifelong health and wellbeing, as well as the importance of identification of childhood adversities within both the social and health fields [27]. Research points to the significance of investing in preventative measures as early as possible in life to support child development [28]. Likewise, Hudziak argues that the developmental cascade from maternal ACEs to child development outcomes seems to start before the pregnancy, accentuating the need for early intervention and support during the pregnancy period [25]. Antenatal care poses an important window of opportunity for such efforts. Despite recognition of the importance of early screening for ACEs [3,29], only a limited body of research has explored the use of the ACE questionnaire [30] in an antenatal care setting [31,32,33,34,35]. Furthermore, existing studies have predominantly used quantitative designs to explore women’s experiences with the questionnaire. Thus, more in-depth research exploring pregnant women’s perspectives is lacking. 

This paper reports on results from ”The Invisibly Vulnerable Study”. The study was initiated to promote systematic screening for ACEs in Danish antenatal care and ensure adequate help for women who, due to the circumstances of their upbringing, may need extra support during pregnancy and after birth. This is the second of two published studies. The first study explored the feasibility and acceptability of the ACE questionnaire and factors affecting its implementation among midwives at three maternity wards in Eastern Denmark. The present study focuses on the pregnant women as the recipients of the ACE questionnaire.

The objective of this study was twofold: to explore pregnant women’s attitudes towards and experiences of the ACE questionnaire, and to assess the relevance of the questionnaire among a population of pregnant women referred to antenatal care levels one and two.

### 1.1. Antenatal Care in Denmark

**Figure 1:**
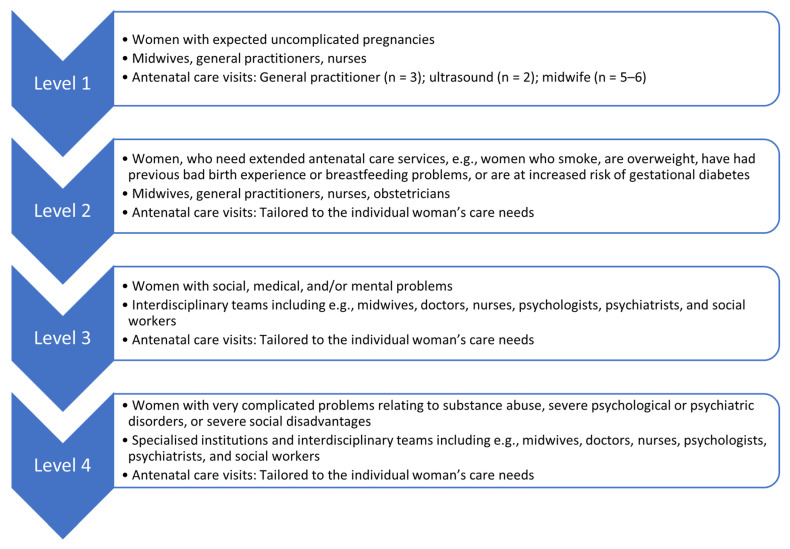
Antenatal care levels in Denmark.

For women who hold a residence permit, antenatal care is publicly funded [36]. Antenatal care is divided into four levels [36]. Figure 1 describes the different antenatal levels in more detail, including the target groups and the professionals involved in the provision of care at the different antenatal care levels. 

In Denmark, most pregnant women are referred to antenatal care levels one and two [37]. For women with expected uncomplicated pregnancies, the midwife is the maternity care provider who the woman sees the most. Women are screened for psychosocial vulnerabilities during the first trimester of the pregnancy. The general practitioner is responsible for confirming the woman’s pregnancy, as well as collecting and assessing their psychological, social, and physical history at the first antenatal care visit. This information is used to refer the woman to the appropriate level of antenatal care [36]. Midwives follow up on women’s history at the first midwifery visit.

### 1.2. The ACE Questionnaire in a Danish Antenatal Care Setting

Between June and October 2021, three hospitals in Eastern Denmark started to include questions on childhood experiences in their existing screening procedures in antenatal care as a quality improvement initiative. The new practice implied that all women assigned to antenatal care levels one and two were asked 10 ACE questions by their midwife at the first or second midwifery visit during the first or second trimester of the pregnancy. The number of births ranged from 1500 to 2400 per year at the three hospitals. Within the framework of this initiative, we designed a feasibility study, which is described in detail under the Materials and Methods section.

The adverse childhood experiences (ACE) questionnaire is an internationally widely used and recognised questionnaire consisting of 10 questions about traumatic experiences in childhood (0–17 years) [38]. The instrument makes it possible to capture different categories of dysfunctional upbringing environments, and it has been found to be a strong predicative measure [3,29,38,39]. The WHO recommends that the tool be widely deployed globally due to its effective predictive value and health-promoting potential [40]. A positive answer to a question adds one point to the respondent’s total score, i.e., scores can be between 0 and 10 points, with ≥4 considered serious [3,30,41]. 

The original ACE questionnaire was described in the Centers for Disease Control Kaiser Permanente adverse childhood experiences study by Felitti and colleagues [38]. It focuses on a person’s experiences from birth to their 18th birthday and is composed of two clusters—one about different types of child maltreatment, and another about household challenges. Instead of questions on different types of childhood adversities, the ACE questions centre around specific situations. For example, a question regarding psychological abuse asks about situations where an adult in the household “often or very often swear at you, insult you, put you down, or humiliate you or act in a way that made you afraid that you might be physically hurt” [30]. In this study, we used a validated version of the ACE questionnaire from the Centers for Disease Control and Prevention and Kaiser Permanente, where question items regarding emotional and physical neglect were added [30]. This questionnaire has been widely used in research [3,29].

A professional forward and backward translation of the questionnaire was carried out [42]. Furthermore, we conducted a cultural translation, with the intention of adapting the questions to Danish midwifery visits and to general conditions in Denmark [43]. A cultural translation is recommended by the WHO to enhance the acceptability and cultural applicability of the questions, as well as the appropriateness of the wording and phrasing [44]. We used a WHO translation guideline regarding another health-related topic for inspiration [45]. We also had an expert group consisting of midwives with antenatal care experiences comment on the Danish version of the questionnaire. The cultural translation led to a slight change in the order of the questions and to some minor modifications of the wording. Sub-items regarding household alcohol and drug abuse were merged into one question about “household substance abuse”, and information on incarceration, which had its own question, was included in a broader question about “loosing contact with a parent”. The final questionnaire included 10 questions on the following items: psychological abuse, domestic violence, physical abuse, sexual abuse, household substance abuse, lost contact to a parent/incarceration, parental separation, household mental illness/attempted suicide, emotional neglect, and physical neglect. 

A project midwife was assigned to assist and monitor the implementation of the ACE questionnaire. The midwives received a whole-day training course on attachment theory, pedagogical theory, ACE research, and how to implement the ACE questionnaire. The midwives also received an implementation manual on how to introduce the questionnaire, ask the ACE questions, and follow up on women’s replies, and they participated in a dialogue meeting where they shared and discussed their implementation experiences. One author (V.d.L.) facilitated the midwives’ training courses and dialogue meetings. 

Most of the midwifery visits were prolonged by 10 minutes to allow adequate time for women to answer the questionnaire and for the midwives to follow up on the women’s replies. Women scoring between one and three ACEs were assessed for their need of an extra midwifery visit. Women scoring four ACEs or more were offered an extra midwifery visit where their ACE replies, current situation, and maternity care needs were discussed. Women who were identified as having mental and/or social problems due to their ACE history were offered referral to antenatal care level three or four, where help from psychologists, psychiatrists, and social workers is available and extra time resources are allocated.

## 2. Materials and Methods

### 2.1. Design

The study was carried out as a feasibility study of the ACE questionnaire in Danish antenatal care. Drawing inspiration from the British Medical Research Council [46], a mixed-methods design was chosen. Bowen and colleagues argue that feasibility studies should be used to assess an initiative’s applicability, including its relevance among its population [47]. At the time of this study, no previous study had uncovered the prevalence of ACEs among pregnant Danish women. Therefore, it was decided to include basic quantitative data in the form of ACE scores for the women who had been screened at the three participating maternity wards. These data were collected from June 2021 to October 2022. The qualitative data consisted of observations of antenatal care visits and in-depth interviews with women. A multimethod approach to the collection of qualitative data was chosen to contribute with different perspectives on how the ACE questionnaire was perceived and experienced by the women. The qualitative data were collected from November 2021 to June 2022. 

### 2.2. Recruitment of Study Participants 

All women completing the ACE questionnaire were asked for permission to submit their ACE scores to the research team by their local midwife. According to the project midwives, nearly all of the women asked consented to share their ACE scores (number not recorded).

To promote heterogeneity in the collection of qualitative data, women were evenly recruited from the three participating maternity wards. For the observations, women were recruited by either the author S.M.K. or the midwife at the antenatal care facility. Inclusion criteria were belonging to level one or two of antenatal care and being born in Denmark. All women invited to participate in an observation agreed to this. The women were invited to participate in an interview by their midwife at the antenatal care facility. Appro-ximately 75% of the women consented to be contacted by phone by the research group. Two authors (H.J. and V.d.L.) recruited women for the interviews. The inclusion criteria for the interviews were the same criteria as for the observations. In addition, as previous feasibility studies of the ACE questionnaire had shown greater client discomfort answering the questionnaire among groups with positive ACE scores compared to groups with negative scores [32,48], it was decided to include women with ACE scores of at least one for the interviews. Twenty women were contacted regarding participation in an interview, and all of these women initially consented to the interview. Five women had an interview scheduled but did not complete it due to being ill, being too busy, or having given birth.

### 2.3. Ethical Considerations 

Women completing the ACE questionnaire were informed about the study’s purpose before giving verbal consent to sharing their ACE score with the research team. Consent to share their ACE score with the research team was documented in the women’s hospital records. Women participating in the observations were informed verbally about the study, and written information was provided prior to their giving written consent to participa-ting in the observations. Finally, women who participated in an interview received verbal and written information about the study before giving written consent to participate in the study. They were also given the opportunity to ask questions before giving consent. All women were informed that they could withdraw from the study at any time, should they wish to do so, and they were guaranteed personal and institutional anonymity. 

Since the ACE questionnaire might induce emotional reactions to previous trauma in some women, extra time was allocated for the midwives to brief and debrief the women. In addition, the midwives’ training course included communication training in facilitating difficult conversations with women affected by the questions. Also, the implementation manual contained recommendations on how to prepare a woman for the questions that followed, and on how to address her experiences. Women who were emotionally affected by the screening process were offered an extra midwifery visit or referred to antenatal care level three. 

Furthermore, due to the intimacy of the topic (adverse childhood experiences), a debriefing was added to the interview, where women’s interview experiences were explored, and they were encouraged to contact the interviewer or their midwife in the event that they found the situation distressing. 

In Denmark, certain types of research projects must be approved by a research ethics committee [49,50]. This applies to clinical trials and studies that involve human biological material. In the Committee Act, Section 14, it is specified that studies that do not involve human biological material should not be reported to the committee, and it is further spe-cified that quality control and quality improvement initiatives should not be reported either [51]. This study, as well as a description of the measures taken to ensure data protection, was reported to the Research, Development, and Data Department, University College Copenhagen (ID number: 21-002). This department acts on behalf of the Danish Data Protection Agency [49].

### 2.4. Data Collection

#### 2.4.1. Observations

To allow for different perspectives of the phenomena [52], structured observations (O) of midwifery visits were performed. These were carried out with the role of the observer as a participant [53], which entailed minimal involvement in the social setting of the midwifery visit. In all, 18 observations were performed across the three maternity wards participating in the study. The observations had a duration of 13–52 min. An observational guide was used to collect the data [54]. The guide collected data on how women interacted with their midwives during the introduction to and completion of the ACE questionnaire, how women responded to their ACE scores and the information provided by their midwives, and the women’s characteristics. Observations were continuously discussed among the authors to enable further exploration of the preliminary findings during the data collection period. Observations were documented as notes at the antenatal care facility and written in full as soon as possible after they had taken place. This was performed by the author S.M.K.

#### 2.4.2. In-Depth Interviews with Women 

The subject of childhood adversities is potentially sensitive. Thus, individual interviews (I) with women were chosen to illuminate women’s attitudes towards and experiences of the ACE questionnaire. Due to the COVID-19 pandemic at the time of the study, and considering the risks related to infection during pregnancy, the interviews were conducted online with camera transmission (*n* = 12), or by phone (*n* = 3). The women selected the date, time, and mode of the interview. 

A semi-structured interview guide was used to collect data [55]. The themes in the guide were centred around women’s pregnancies, social support/situation at home, childhood adversities and potential health consequences of these events, interactions with the midwife regarding the ACE questionnaire, and expectations of parenthood. Follow-up questions enabled the women to elaborate on their initial replies and allowed for flexibility in the collection of data [56]. As the analysis of the data started before the data collection ended, this allowed for further exploration of preliminary themes towards the end of the data collection period. The average interview duration was one hour and two minutes. All interviews were audio-recorded and transcribed verbatim. The interviews were conducted by two authors (H.J. and V.d.L.).

### 2.5. Data Analysis

Descriptive data on women’s ACE scores, including the total numbers for each ACE category and the women’s summative ACE scores, were reported by the midwives at the antenatal care facilities. All data were collected anonymously and on an individual level. Data were checked, controlled, and combined with double entry by one author (E.R.). If differences occurred between the midwives’ scoring and the summative ACE scores, the recorded summative ACE scores were used. Descriptive and summative figures are presented (Figures 3 and 4). ACE scores of 0 were considered to represent a low risk of negative effects of childhood trauma, ACE scores of 1–3 as moderate risk, and ACE scores of 4–10 as high risk [3].

**Figure 2:**
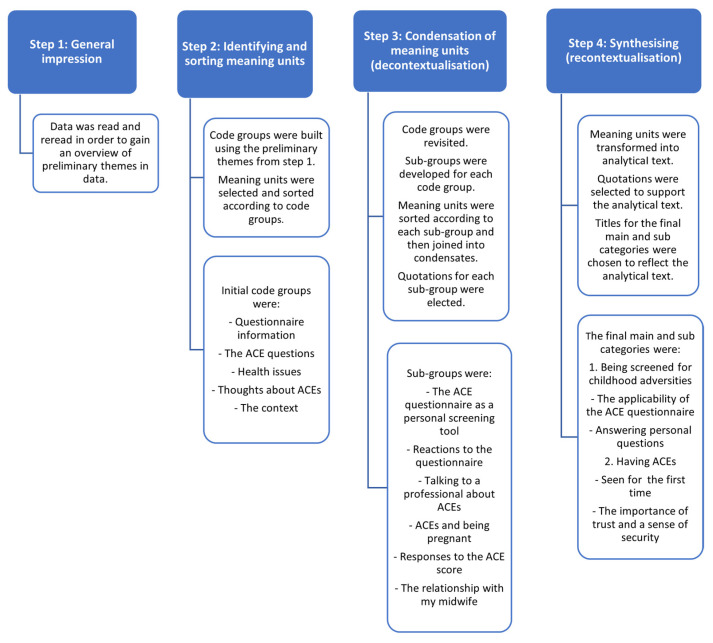
The four analysis steps.

NVivo [56] was used to store and manage the qualitative data from the observations and interviews. Data were Analysed using systematic text condensation [57]. This method consists of four steps: (1) total impression, (2) identifying and sorting meaning units, (3) condensation of units and themes, and (4) synthesising. Observations and interviews were analysed with the same codes and then merged in step three of the text-condensation process. Two authors (H.J. and V.d.L.) undertook analysis step one, and H.J. undertook ana-lysis step two. The remaining analysis process was discussed among the authors to ensure that the categories and subcategories were grounded in the entire dataset. Figure 2 describes the analysis process in more detail.

## 3. Results

### 3.1. Women’s ACE Scores

**Figure 3:**
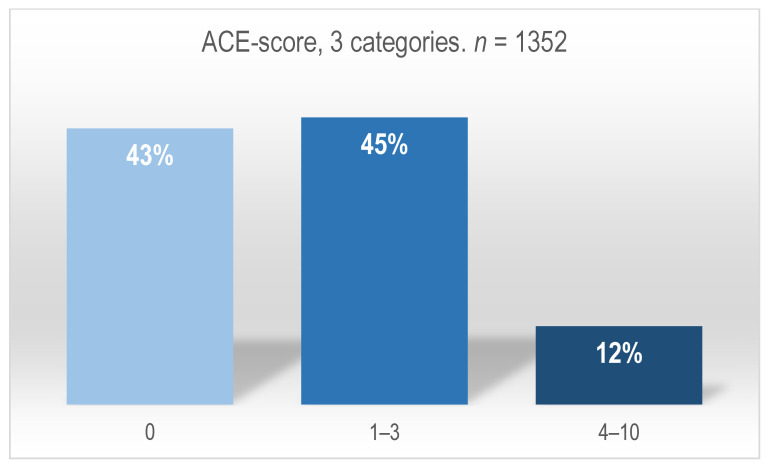
Adverse childhood experiences distribution according to the three ACE categories.

**Figure 4:**
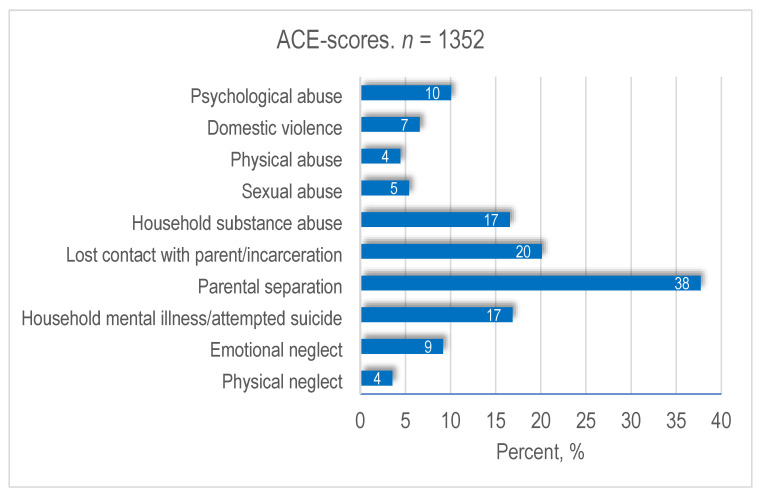
The distribution of each adverse childhood experiences item.

A total of 1352 women had their ACE scores collected at the antenatal visits. Figure 3 shows the three ACE categories. Figure 4 shows the distribution of each of the 10 ACE items in percentages. 

As shown in Figure 3, 43% (*n* = 582) scored negative on having adverse childhood experiences (ACE 0), 45% of the women had an ACE score between 1 and 3 (*n* = 605), and 12% had a score from 4 to 10 (*n* = 165).

In Figure 4, we can see that the individual person may score on more than one item, which explains why the sum of percentages exceeds 100. That means, for example, that 10% of the total sample reported being subjected to “psychological abuse”, 7% to “domestic violence”, and so forth. The ACE item that scored highest, “Parental separation”, was reported by 38% of the women. Seventeen percent reported having an upbringing with either household substance abuse or mental illness/attempted suicide in the family. Five percent reported being subjected to sexual abuse.

### 3.2. Women’s Characteristics

A total of 18 women participated in the observations of the midwifery visits. They were between 15 and 23 weeks pregnant. Six women were expecting their first child, ten their second, and two their third or fourth. 

A total of 15 women participated in the in-depth interviews. They were between 20 and 39 years old (average: 29 years). Five women were expecting their first child, eight their second, and two their third. The women were between 23 and 39 weeks pregnant at the time of the interview. Six women had an ACE score below four points and nine had an ACE score between four and eight. Two women had public school, five college, and eight university graduation as their highest educational level. All women were cohabiting with a male partner.

### 3.3. Being Screened for Childhood Adversities

Analysis of the qualitative data resulted in two main categories: The first main category was being screened for childhood adversities, which describes women’s experiences of being introduced to and completing the ACE questionnaire. 

#### 3.3.1. The Applicability of the ACE Questionnaire

Observations and interviews both indicated that the women generally consented to answering the ACE questionnaire. Interviews with the women revealed that a few women had initially been surprised that their midwife asked about their childhood at the visit. Generally, the women reported the allocated timeframe for answering the questionnaire as adequate. Likewise, the observations showed that the 10 minutes allocated for being introduced to, answering, and discussing the ACE questionnaire was sufficient in most of the midwifery visits observed. Only one woman felt that she had needed more time to answer the questions. Furthermore, observations and interviews both suggested that the women were overall able to answer the ACE questions without needing them to be further explained:

“I found the ACE questions to be phrased well.”

(Pregnant woman, I11)

Nevertheless, a few women did find it difficult to determine whether they should answer yes or no to an ACE question. Their doubts pertained to when a caregiver’s behaviour should be labelled as problematic or as a part of one’s upbringing, as shown by the following observation:

“When the woman is asked about physical abuse directed towards her, she initially answers no. After, she tells the midwife that her mother had once slapped her in the face. It happened during a period where her mother was very stressed…She didn’t think it was okay and wants to know if the episode counts as physical abuse. The midwife tells the woman that this depends on the woman’s estimate of the situation… The women decides that she doesn’t think it was physical abuse.”

(O1)

Midwives’ introductions to the ACE questionnaire mattered for the women’s willingness to share their childhood experiences. The fact that the questionnaire was introduced as an initiative seeking to improve antenatal care for women with childhood adversities enhanced the women’s motivation to answer the questionnaire. The women also appreciated that the ACE questionnaire was universally implemented for all women in levels one and two of antenatal care, because this decreased the potential stigma related to the individual ACE screening.

The women explained that they found the ACE questions suitable for addressing a range of potential childhood adversities. According to the women, a major benefit of the ACE questionnaire was its ability to identify potentially vulnerable pregnant women. In particular, the importance of childhood adversities for parenting skills was mentioned by the women:

“…she (the midwife) explained the background for the initiative…you know that your own upbringing matters for how you become a family and a parent. They (the midwives) wanted to help earlier, identify women earlier, to support women in need of help…I thought it made really good sense…”

(Pregnant woman, I1)

“…I think it (the ACE questionnaire) makes sense…it matters for the future, for your children, the way you interact with your own child…we are all different….some women are very affected by what they have experienced.”

(Pregnant woman, I8)

Some women described how they had discussed their ACE scores with their partner. These discussions had centred around potential risks when becoming a parent:

“…you don’t think it (your ACEs) can affect you…but when your life is about to change drastically (becoming a parent), it makes sense that it can affect your coping mechanisms. He (my partner) thought it was fine that my childhood was addressed… also that the ACE score became part of my hospital record, so that my upbringing is part of my history.”

(Pregnant woman, I4)

#### 3.3.2. Answering Personal Questions

All of the women described they were aware of the fact that their upbringing had been traumatic in one or more respects prior to answering the questionnaire:

“The woman tells the midwife that her mother had been an alcoholic…As a teenager she had carried many responsibilities at home…The midwife wants to know how the woman feels about her (ACE) screening. The woman answers that she feels fine… She explains that she is very aware of the challenges she endured during her upbringing.”

(O11)

Another woman explained:

“…it can sound extreme, but for me it’s not. I am very aware that this is not the environment a child should grow up in…I think I have just gotten used to it, that this was how it was…That’s why it doesn’t affect me to talk about it…The fact that I scored high (on the ACE questionnaire)…that my score was in the bad end of the scale, didn’t surprise me at all.”

(Pregnant woman, I15)

Our observations showed that the women were often asked by their midwives how they felt after answering the ACE questions. The women generally responded positively to the screening process, as demonstrated by the following quotes:

“The woman tells the midwife that her parents got divorced when she was in her early teens…Three years later she lost her father to cancer…After answering the ten ACE questions, the midwife asks the woman: ‘How was it for you?’. The woman answers: ‘It was fine, I am glad that you asked’.”

(O16)

“As a child she had witnessed domestic violence…Her father had been sentenced to prison, where he later died…As a teenager, she had been removed from home by the social authorities due to her mother’s depression and alcohol abuse…It had been very traumatic… The midwife asks her how she feels after answering the ACE questions. She replies that she feels ‘okay’. She describes that she has used a lot of time and energy as an adult to ‘reach the other side’.”

(O10)

At the same time, the women overall seemed to agree that the ACE questions were intimate. One woman explained how sharing her childhood adversities with another person, who was more neutral regarding her upbringing, was easier for her than sharing these experiences with someone she knew:

“…There are many people, who have had a problematic childhood, so have I…I don’t mind sharing, especially with people I don’t know. Actually, it’s easier for me to talk about my childhood with people I don’t know than it is to talk about it with people who are close to me.”

(Pregnant woman, I14)

Several women described how their midwife had advised them about the nature of the ACE questions before posing them. Although the women were generally motivated to answer the questions, and most of the women described the process of answering the questionnaire as a positive experience, some women also described the ACE questions as emotionally demanding. In particular, questions that induced feelings of embarrassment or portrayed women’s caregivers as indifferent were described as affecting the women:

“It was a question related to food…whether there was food available. I sat thinking: ‘Should I say this?’ You sit there embarrassed and ashamed over the environment you grew up in…My mum used most of her time telling me off…she didn’t prepare lunch for me and my brother… so we often went to school without food. ‘If you are hungry, then go make yourself some food.’ Well, I would if there was any food in the fridge, but there wasn’t. It felt like I ratted her (my mother) out. On the other hand, that was how it (my childhood) was.”

(Pregnant woman, I6)

“It’s hard (the questions)…I am thinking, how could you do that to your own child…put your own needs before your child’s…the question about whether I felt loved really resonated with me, because for many, many years I would run away from home…if I could just die a little, just a little, then maybe I could find out if they (my parents) ever loved me.”

(Pregnant woman I15)

A few women who had scored high on the ACE questionnaire highlighted that there were other women who were much more affected by their childhood adversities than they were. A woman who had experienced psychological and physical abuse by her stepfather and had been affected by these experiences as an adult explained the following:

“…I think there are people, who come from the same world (childhood abuse) but have had it ten times worse than me. Compared to the average person in Denmark, I expected to score on it (the ACE questionnaire) because there have been situations, which have affected me a lot…”

(Pregnant woman, I14)

### 3.4. Having ACEs 

This category illuminates women’s perspectives related to screening positive for ACEs, as well as the significance of their relationship with their midwife for their screening experiences. 

#### 3.4.1. Seen for the First Time 

None of the women in this study had previously answered the ACE questionnaire. Some of the women had previously talked to social workers or psychologists about the circumstances surrounding their upbringing. However, several women who described having experienced multiple ACEs had not had these experiences addressed by a professional during their childhood or adolescence, nor had they had any help from their family or friends in coping with their circumstances. As a result, they had largely dealt with their traumatic experiences on their own. One woman, who had experienced psychological and physical abuse as well as emotional neglect from her mother and father, described how, as a child, she had kept her experiences to herself. Her schoolteacher had been aware of the fact that she carried many responsibilities at home. However, she had not told her teacher about her abuse and neglect, due to fear of being monitored by the authorities — a fear that her parents had cultivated:

“If you tell anyone, you will have a social worker assigned to your case. They (the social authorities) will constantly be keeping an eye on you…many things in your life will no longer be possible. This was what I was told during my childhood.”

(Pregnant woman, I9)

Discussing adverse childhood experiences with healthcare professionals was also very rare among the women. Several women described how the midwife was the first professional with whom they had ever shared their childhood experiences. One woman described how both she and her partner had experienced a traumatic upbringing. She explained that her partner had a record with the social authorities, but she did not. Thus, the traumatic circumstances pertaining to her upbringing had not previously been identified:

“I am looking at the ACE questions and to be honest, my feelings told me they (the questions) made a lot of sense. Answering them, I didn’t feel overrun…deep down I thought it’s a really good thing. For me it is very important that they (the maternity care providers) focus on those of us, who do not have a previous re-cord with the social authorities, so that we can get help, if we need it….”

(Pregnant woman I11)

Another woman described how her traumatic upbringing had not been addressed by the midwife during her first pregnancy. At the time, she had thought of her childhood as a finished phase of her past. However, after the birth of her first baby, she had been more emotionally affected by her childhood experiences than she had anticipated. When the ACE questions were introduced by her midwife during her second pregnancy, she had used the questionnaire as an opportunity to talk to her midwife about her upbringing, as well as about how she could prepare herself emotionally for the birth of her second child:

“As a first-time mother…I wasn’t aware that my feelings during childhood would come back to me like they did. I thought this chapter (of my life) was over, but when you are holding your gold (the baby) in your arms, something happens to you due to your bad childhood…It’s important to be more prepared…that you can talk about it (with your midwife)…there is a need for more focus on pregnant women who are vulnerable…”

(Pregnant woman, I10)

A few women described how it was difficult for them to express how they felt, and that they were unlikely to inform others about their traumatic childhood experiences unless they were asked directly. One woman, who described herself as an introvert, explained:

“I am much more of a closed book…you can ask me anything, that’s not a problem, I just won’t tell it on my own.”

(Pregnant woman, I1)

#### 3.4.2. The Importance of Trust and a Sense of Security 

The woman–midwife relationship was pivotal for how the women perceived their experiences with the ACE questionnaire. While the interviews with the women suggested that they themselves were generally not surprised about their ACE scores, a few women did explain that they had been affected by the fact that their midwife had seemed surprised about their ACE replies. One woman described how her midwife’s facial expression had changed when she responded to the ACE questionnaire:

“…it’s not very comfortable to sit there and tell what you were exposed to as a child, because the other person (the midwife) gets that weird expression in her face, probably because it is unexpected…she has another impression of who you are, and then all of a sudden some things are put on the table, which are not so pleasant to hear…”

(Pregnant woman, I9)

The timing of the questionnaire was another factor mentioned by the women. One woman explained how having the questionnaire presented during the first midwifery visit meant that she had not had time to establish a relationship with the midwife:

“… if you are diving into these things (my upbringing)…then it needs to be with someone you feel safe with, this is very complex…I found it overwhelming, you have just met the person…if it had been the second or third time, then maybe it would have been different.”

(Pregnant woman, I10)

A few women also explained how their scores had induced concerns about how the midwife would perceive them after they had completed the ACE questionnaire. In particular, concerns related to their motherhood abilities and risks of becoming a person of interest with the social authorities were described by these women:

“…it’s that feeling of not being good enough…what they think about me, she (the midwife) probably thinks, that I will become a really bad mother because of my terrible childhood, that I will be passing that on to my child…you can feel like you are being judged, but you are not really…my midwife was very open-minded…”

(Pregnant woman, I1)

“Of course, it (your traumatic upbringing) is a little intimidating to express out loud, because you don’t want to feel like or present yourself as a victim and someone unable to take care of your child, just because you come from something, which was not picture perfect.”

(Pregnant woman, I11)

Trust and feeling secure were key elements for how the women felt during their interactions with the midwife. The safer and more secure the women felt at the midwifery visits, the more willing they were to provide detailed descriptions of their upbringing and to discuss the possibilities of extended antenatal care. Not feeling judged by their midwife was described as extremely important. One woman explained how she had been assured by her midwife that although she had an ACE score of four, she would not be referred to a psychiatrist. Being reassured had made the woman more motivated to discuss her care needs with the midwife:

“I thought it was nice to sit with my midwife and follow up on my ACE score. She told me, that due to my answers, I landed in the vulnerable category. She assured me that they wouldn’t be calling the psych ward. I knew all kinds of initiatives wouldn’t be started up because I scored four…instead the ACE questionnaire was a way to identify pregnant women, who could be in need of extra midwifery visits.”

(Pregnant woman, I4)

Another woman described how it had been very important for her that her midwife had been unprejudiced towards her ACE replies:

“I felt really safe in the company of my midwife…she is open minded and she doesn’t have that judgmental look…which was nice…being looked down on…it’s not my fault…it’s your parents job to provide a good childhood for you…but, as child, you can feel terrible and ashamed over that fact that your childhood was so bad…The (ACE) questions created a room for talking…about the issues I needed to talk about.”

(Pregnant woman, I6)

Finally, continuity of carer was described as a prerequisite for following up on the women’s ACE scores at a subsequent midwifery visit and planning antenatal care. A woman described that although she had scored 4 on the ACE questionnaire, her score had not been further addressed by anyone due to seeing a new midwife at every visit:

“I haven’t seen the same midwife any of the three visits. I’ve felt like I have had to start over every time…Next visit I am apparently seeing a new one again…”

(Pregnant woman, I14)

## 4. Discussion

The participating women generally found the ACE questionnaire to be a relevant screening tool, indicating high acceptability of the questionnaire. These findings are aligned with other studies showing high acceptability of the ACE questionnaire among women before and after birth [1,48,58]. A study by Flanagan and colleagues found that the majority of the women in antenatal care agreed that ACEs could have lifelong health consequences and that they were satisfied with the screening process [32]. Nearly all existing studies have used quantitative methods to explore the acceptability of the ACE questionnaire among pregnant women. The in-depth interviews in the present study allowed for a more comprehensive understanding of why the women found the ACE questionnaire to be an acceptable screening method. Women in this study highlighted the potential impact of adverse childhood experiences on their parenting skills as being especially important. This finding suggests that women are open to disclosing possible vulnerabilities caused by traumatic circumstances of their upbringing because they are concerned that these vulnerabilities may affect the wellbeing of their offspring. Literature from the field of developmental psychology has pointed to pregnancy as a transitional period in life, where women reflect more on the significance of their past and values for their future parenthood [59]. Hence, the pregnancy period presents a critical window for identification and intervention. As argued by Hudziak, maternal adversity, regardless of socioeconomic background, needs to be asked about and addressed to prevent intergenerational transmission of parenting skills and the risks of affecting subsequent child development [25].

Overall, the women described the time available to answer the questionnaire as sufficient and the ACE questions as comprehensible. In addition, the fact that the questionnaire was universally implemented seemed to matter to the women, as this contributed to legitimising the midwives’ use of the questionnaire. At the same time, some of the women described replying to some of the ACE questions as emotionally demanding, as well as feeling embarrassed when affirming abuse, household dysfunction, or neglect. Discomfort related to disclosing childhood trauma has been addressed in previous studies. A study by Mersky and colleagues found that vulnerable pregnant women’s and mothers’ discomfort varied according to the specific ACE question [48]. Parental divorce induced the least discomfort, while sexual abuse induced the most. A study by Flanagan and colleagues found that pregnant women with positive ACE scores were more likely to feel discomfort than pregnant women with negative ACE scores [32]. This study only included women with positive ACE scores. Our findings suggest that rather than feeling uncomfortable, some of the women described being emotionally affected by the ACE questions, especially if they had experienced these adversities themselves. Moreover, emotional reactions to specific questions did not appear to affect these women’s perceptions of the ACE questionnaire as a relevant screening tool. 

Several women described how their childhood trauma had not previously been addressed by a social worker or a healthcare professional. As a result, the midwife was the first professional with whom these women had talked about the circumstances of their upbringing. These findings are supported by other studies documenting that within the field of maternal care and childcare, many cases of childhood trauma in women are unrecognised prior to screening for adverse childhood experiences [1,32]. Quantitative data in this study showed that 43% of the women reported zero ACEs, 45% reported between one and three ACEs, and 12% reported four or more ACEs. This prevalence and distribution of ACEs reflects the results of other studies. Findings from a Canadian cohort study documented a prevalence of 37.5% of pregnant women with an ACE score of zero, 47.3% with an ACE score of one to three, and 14.7% with an ACE score of four or more [17]. A Swedish survey study documented a prevalence of 41.6% of pregnant women with an ACE score of zero, 58.6% with an ACE score of at least one, and 7% with an ACE score of five or higher [16]. In the Swedish study, the prevalence of ACEs was measured in a population that included women with previous psychiatric disorders. In Denmark, these women are referred to antenatal care level three or four. In the Danish study population, all women were attending antenatal care level one or two—levels that provide antenatal care for women who are not identified as psychosocially vulnerable. Some of the women described entering antenatal care with a history of ACE-induced mental health problems. Despite these health problems, these women were still referred to antenatal care levels one and two, possibly due to lack of information in the women’s antenatal records. The ACE screening helped to explain the causes of these health issues. For example, the quantitative data showed that 17% of the women with a positive ACE score had grown up with household substance abuse, and 17% had grown up with household mental illness/attempted suicide—clinical knowledge that is important when assessing these women’s care needs. Also, although research so far has documented higher health risks in individuals with four ACEs or more [3], the qualitative data from this study indicated that the potential impact of an ACE depended on the unique experience and whether the woman had previously been identified and received help in coping with these experiences. Thus, for some women, an ACE score of one may have a more profound health impact and require more antenatal care support than an ACE score of four for others. 

Our findings also showed that the women’s total ACE scores were anticipated by the women. At the same time, some women expressed concern that a positive ACE score could potentially lead to midwives’ doubts about their motherhood abilities, as well as risks of being reported to the social authorities. These findings mirror those of previous Danish qualitative research showing that adult daughters of alcoholic parents can have concerns of the potential stigma associated with disclosing childhood trauma in antenatal care [60]. Feelings of stigma are important, because they may lead to self-doubt and reluctance to ask for help, emphasising the importance of training maternity care providers to have a non-judgmental approach to women’s ACE history. In addition, our findings showed that some women who scored high on the ACE questionnaire described other women as being worse off than themselves, despite being affected by the circumstances of their upbringing. Related findings are presented in a study of the ACE questionnaire in home-visiting programs in Wales, where mothers displayed a willingness to expose their ACEs but were more hesitant to discuss their ACEs in detail, as well as to receive extra care due to these experiences [1]. Interestingly, in the study from Wales, 40% of the women had not previously discussed the circumstances of their upbringing with a healthcare professional and, thus, had not previously received healthcare support for their possible trauma. 

Finally, experiences of being screened for ACEs were highly dependent on women’s relationship with their midwife. It was important for the women to establish a relationship with the midwife before being screened, suggesting that the timing of the questionnaire during the visit mattered for the women’s experiences. In this study, the ACE questionnaire was mainly implemented at the first midwifery visit to promote early identification of ACEs and to make a plan for antenatal care based on the individual women’s needs. However, some women may need more time to establish a trusting relationship with their midwife. As shown in the study, feeling secure in their relationship with the midwife affected the women’s willingness to disclose a more detailed description of the circumstances of their upbringing. Although some Danish maternity wards provide caseload midwifery (i.e., maternity care provision by a small team of midwives) to a proportion of women, most pregnant women in antenatal care levels one and two receive standard care [37]. In this study, women noted how discontinuity of care affected their communication with the midwife about their care needs. Related findings are presented in a recent literature review showing that continuity of care can enhance women’s perceptions of being known by the midwife and can increase the provision of personalised care [61]. These considerations may be especially important, when screening for experiences of a more sensitive nature, such as adversities during women’s childhood. A few women had noted the midwives’ facial expressions and surprise when they had scored positive on the ACE questionnaire. A study investigating provider and client discomfort with the ACE questionnaire has shown a positive association between provider discomfort and client discomfort [48], suggesting that care providers’ reactions to patients’ replies affect the patients’ experiences of the screening process. Flanagan and colleagues highlighted the significance of training to increase provider confidence with the ACE questionnaire [32]. In this study, the midwives were offered a whole-day training course on how to implement the ACE questionnaire, as well as a dialogue meeting where they shared their implementation experiences. However, as the ACE questionnaire was a novel screening tool in Danish antenatal care, this may have affected the interactions between the women and the midwives. 

### Main Limitations and Strengths

Due to previous research showing greater client discomfort answering the questionnaire among groups with positive ACE scores compared to groups with negative scores [32,48], we chose to include women with an ACE score of at least one for the interviews. Thus, these findings do not reflect the attitudes and experiences of women who with negative ACE scores. Exploring this group’s experiences could have further nuanced our findings regarding the feasibility and acceptability of the questionnaire. Also, the women were recruited from antenatal care levels one and two, and women with more complex psychosocial issues attending antenatal care levels three and four might have responded differently to the questionnaire. Furthermore, all of the women were living with a male partner. A previous qualitative study has shown that, for women with adverse childhood experiences, their partner can be an important source of emotional support during the pregnancy period [60]. Therefore, women participating in the interviews may have been more resourceful than the overall study population. Also, all of the women in the study were born in Denmark. Feasibility and pilot studies of the ACE questionnaire have so far been undertaken in Western countries [29,34]. However, the cultural acceptability of the ACE questionnaire may vary between different countries. Finally, online interviews, and especially telephone interviews, can decrease attention to non-verbal cues during the interview. 

The major strengths of this study include the triangulation of data sources, data collectors, and data analysts. This increases the credibility of the findings [52]. In addition, data were collected at three maternity wards and five affiliated antenatal care facilities, which added to the heterogeneity of the sample. This enhances the transferability of the findings to similar organisations of maternity care. Also, data were collected and analysed throughout the implementation period. This meant that the women were interviewed when the ACE questionnaire was new in practice, as well as when the midwives had accumulated more experience with the questionnaire. In addition, the continuous collection and analysis of data allowed for the assessment of sufficient information power in the data [57].

## 5. Conclusions

The prevalence of ACEs in the study population was high. Fifty-seven percent of the women had a positive ACE score, and twelve percent had a score of four or more. Parental separation (38%), lost contact with a parent/incarceration (20%), household substance abuse (17%), and household mental illness/attempted suicide (17%) were the most common categories reported by the women.

Despite some women being emotionally affected by the ACE questions, the women overall found the ACE questionnaire to be a relevant and acceptable screening method. The midwifery visit was the first time that several of the women had discussed their adverse childhood experiences with a healthcare professional. Trust and feeling secure in their relationship with the midwife were the factors that most affected the women’s screening experiences. 

Although the women in this study were offered extra midwifery visits and referral to extended antenatal care services, the study’s findings call for future research on appropriate antenatal interventions for women who, due to the traumatic circumstances of their upbringing, are vulnerable and, thus, need more targeted interventions. Future research could also investigate whether a combination of an ACE screening for women and their partners during the pregnancy period along with initiatives aimed at cultivating parenting skills, may affect the future health and wellbeing of the child.

## Data Availability

According to the General Data Protection Regulation, the qualitative data are confidential and cannot be provided.
